# Final whistle or beyond? Temporal dynamics of anxiety and psychological recovery in women’s soccer

**DOI:** 10.3389/fpsyg.2025.1697362

**Published:** 2025-12-18

**Authors:** Buse Sulu, Şeval Kayğusuz, Erdem Çakaloğlu

**Affiliations:** 1 Faculty of Sport Sciences, Istanbul Esenyurt University, Istanbul, Türkiye; 2 Faculty of Sport Sciences, Istanbul Gedik University, Istanbul, Türkiye; 3 Faculty of Sport Sciences, Marmara University, Istanbul, Türkiye

**Keywords:** anxiety, emotions, perceived difficulty, psychological recovery, women’s soccer

## Abstract

Anxiety and emotions have been identified as fundamental determinants of psychological recovery. While positive emotions and self-confidence facilitate athletes’ ability to regain balance and recover psychologically in the face of challenges, negative emotions together with cognitive and somatic anxiety may hinder this process. In this regard, it is important to examine psychological recovery in conjunction with anxiety and emotions to capture its dynamic nature. The aim of this study was to investigate the role of emotions and anxiety in athletes’ psychological recovery and to evaluate their seasonal changes. The sample consisted of 20 female soccer players from Kastamonu Gücü Sports Club, competing in the Women’s 2nd League Group C in the 2023–2024 season. Analyses were conducted using R (version 4.4.1; R Core Team, 2024) with linear mixed-effects models (LMM). The models included both fixed effects and random intercepts for athletes. Analyses were performed with the *lme4* and *lmerTest* packages, while model fit indices and explained variance were calculated using the *MuMIn and performance* packages; model assumptions were tested and VIF values were reported. The findings revealed that psychological recovery was supported by self-confidence and positive emotions, whereas cognitive/somatic anxiety, anticipated match difficulty, and negative emotions weakened it. In addition, a decline in recovery was observed as the season progressed. The models explained a large proportion of the variance in psychological recovery (R^2^m ≈ 0.60–0.65). The contribution of individual differences was limited (ICC ≈ 0.11–0.14), suggesting that shared seasonal processes were more influential than individual-level factors. In conclusion, psychological recovery is a dynamic process shaped by both protective (self-confidence, positive emotions) and risk-related (anxiety, anticipated match difficulty, negative emotions) factors. The seasonal decline underscores the importance of systematically integrating practices to support psychological recovery to sustain performance throughout the season.

## 1 Introduction

Soccer, recognized as one of the most demanding and widely followed team sports, consistently places athletes under intense pressure to maintain peak performance across successive competitions (Madsen et al., 2022). Beyond physical conditioning, the capacity to regulate psychological states is acknowledged as a critical determinant shaping both immediate performance outcomes and the long-term development of athletes. While much of the existing research has focused on male soccer players, there is a growing need to examine these psychological processes within the context of women’s soccer, where physiological, tactical, and psychosocial dynamics may differ. Previous studies examining psychological attributes in soccer have predominantly focused on anxiety, identifying it as a significant factor that can influence performance both positively and negatively (Cox, 1998; Reilly et al., 2000; Tossici et al., 2024). Since anxiety is closely linked to the anticipated consequences of success and failure, it plays a decisive role in elite soccer, where research has shown that it can significantly shape future success and performance trajectories (Höner and Feichtinger, 2016; Pelka et al., 2017; Harrison et al., 2021; Sánchez-Ruiz et al., 2025). Building on this, it is essential to consider gender-related differences. In recent years, the growing interest in women’s soccer has played an important role in safeguarding the health and well-being of professional female soccer players, as well as in optimizing their performance (FIFA, 2019; Randell et al., 2021). Female athletes generally report higher levels of anxiety and emotional reactivity, indicating that they may be more sensitive to performance pressures and that these factors can meaningfully influence their competitive outcomes (Martens et al., 1990b; Jones et al., 2005; Grossbard et al., 2009). Such gender-specific patterns suggest that anxiety may have particularly pronounced implications for women’s competitive outcomes, underscoring the importance of addressing these dynamics within the context of women’s soccer.

In the moments immediately preceding competition, state anxiety levels may exert a significant influence on performance outcomes by shaping athletes’ cognitive appraisals and physiological responses (Martens et al., 1990b), with these effects being further moderated by athletes’ perceived match difficulty. Among these cognitive appraisals, perceived match difficulty has been shown to hinder performance in the sport context (Davis et al., 2021). As the tasks performed during competition are perceived as more difficult, the athletes’ a performance may become constrained (Salom Martorell et al., 2021). Evidence from athlete evaluations indicates that increased perceived difficulty may lead to poorer performance, reduced self-confidence, and heightened state anxiety (Mabweazara et al., 2017; Rocha and Osorio, 2018; Barbosa-Granados et al., 2022). Given that anxiety can manifest through different cognitive and physiological responses affecting performance, the Multidimensional Anxiety Theory (Martens et al., 1990a) provides a valuable framework to explain how these distinct components influence athletic performance. According to this theory, competitive state anxiety comprises two distinct components: cognitive anxiety (mental concerns such as worry and negative expectations) and somatic anxiety (physiological activation such as tension or increased heart rate). The theory posits that cognitive anxiety has a negative linear relationship with performance, as excessive worry can disrupt attentional focus and decision-making processes (Eysenck et al., 2007). In contrast, somatic anxiety follows an inverted-U relationship with performance, suggesting that moderate levels of physiological activation may facilitate, while very high levels may impair, performance (Craft et al., 2003). In addition, the model incorporates self-confidence as a distinct dimension, which functions as a protective factor that can mitigate the detrimental effects of both cognitive and somatic anxiety on performance (Woodman and Hardy, 2003). Building upon this, it becomes essential to explore the role of emotions, including anxiety, and how they interact to shape athletes’ psychological experiences and performance outcomes.

According to Fox (2008), emotion is defined as a dynamic process that includes experiential, cognitive, motivational, expressive, physiological, neurological and behavioral components and serves to reorganize the cognitive system. Gross (1998) defines emotion as a broad concept encompassing various phenomena that can be evaluated along the positive–negative dimension. Positive emotions, such as happiness and confidence, can enhance athletes’ attentional focus, motivation, and decision-making capacities (Janelle et al., 2020). Conversely, negative emotions, including anxiety and fear, may contribute to attentional lapses, increased muscular tension, and suboptimal performance outcomes (Hanin, 2000). Nonetheless, negative emotions do not invariably impair performance; in certain contexts, athletes may deliberately channel these emotions to generate motivational momentum (Tamir et al., 2008). Moreover, it is essential to consider that the influence of emotions is not static; rather, their effects evolve dynamically over time, shaping athletes’ psychological states and performance trajectories within a temporal framework (Lane and Terry, 2000; Lazarus, 2000; Tamminen and Gaudreau, 2014). The Individual Zones of Optimal Functioning (IZOF) theory proposed by Hanin (2000) suggests that athletes’ emotional states prior to competition play a critical role in determining their performance outcomes. Should pre-competitive emotional states consistently demonstrate an impact on athletic performance (Hanin, 2000; Cerin, 2003), it follows that emotions are prone to fluctuation throughout the course of competition (Cerin et al., 2000). The majority of research has concentrated on descriptive analyses, relying on retrospective self-reports (Martinent and Ferrand, 2009; Martinent et al., 2012) or examining the relationship between pre-game emotional states and performance outcomes (Hanin, 2000). However, recent research on the temporal dynamics of emotions primarily focuses on the characterization of pre-competition, during-competition, and post-competition states. Post-competition emotional states play a crucial role in the psychological recovery process, influencing athletes’ ability to restore both mental and physical resources necessary for subsequent performance (Fletcher and Scott, 2010; Kellmann, 2010). In this regard, recovery should be conceptualized not merely as a physiological process but also as a psychological phenomenon that shapes athletes’ overall adaptation and readiness for future demands. The psychological dimension of recovery involves the improvement of subjective emotional states, relaxation, a heightened sense of well-being, and the restoration of a positive mood (Kellmann and Kallus, 1999).

Beyond affective restoration, recovery further entails the replenishment of cognitive resources, vigor and energy, all of which are fundamental for sustaining attentional focus and cognitive flexibility in competitive contexts (Ryan and Frederick, 1997; Kellmann, 2010). Equally important is psychological detachment, referring to the capacity to mentally disengage from sport-related stressors and performance demands, thereby interrupting rumination and enabling athletes to experience genuine recovery (Sonnentag and Fritz, 2007). Finally, recovery extends beyond the neutralization of strain to encompass a positive shift toward flourishing, wherein athletes not only regain energy and well-being but also experience renewed purpose, growth, and optimal functioning (Kayğusuz, 2024). From this perspective, recovery is best conceptualized as a dynamic process that fosters resilience and long-term psychological thriving rather than a mere return to baseline (Kellmann and Kallus, 1999; Keyes, 2002; Lundqvist, 2011; Seligman, 2011).

In recent years, the rapid growth of women’s soccer worldwide has attracted increasing professional and media attention to female athletes. Notably, the Fédération Internationale de Football Association (FIFA) aims to increase the number of registered female players from 13.3 million in 2019 to 60 million by 2026 (FIFA, 2019). This expansion has not only enhanced the visibility and competitiveness of the sport but has also intensified the psychological demands placed on players. Consequently, performance-related anxiety and the need for effective psychological recovery strategies have become increasingly salient topics in women’s soccer (Randell et al., 2021; Lovell and Okholm Kryger, 2025). Taken together, these insights underscore the pivotal role of emotion regulation, anxiety, and recovery strategies in shaping athletes’ performance and long-term psychological recovery, while emphasizing the value of longitudinal monitoring to capture dynamic changes. This study aims to examine the role of competitive state anxiety, positive and negative emotions, and perceived match difficulty in female soccer players’ psychological recovery, as well as to evaluate the temporal evolution of recovery across the season.

## 2 Materials and methods

### 2.1 Participants

Ethical approval for this study was obtained from the Health Sciences Ethics Committee of Istanbul Gedik University (Approval Code: E-11470191-050.04-2025.173340.39). This study involves the longitudinal monitoring of players from Kastamonu Gücü Sports Club (Turkey), competing in the Women’s 2nd Soccer League Group C in the 2023–2024 season. Repeated measurements were taken from each participant throughout the entire 14-week competitive season. Participation in the study was voluntary, and all athletes provided informed consent prior to data collection. Four of the 24 athletes in the team roster at the start of the season were unable to participate regularly in the data collection process in the following weeks of the season due to injuries, transfers/withdrawals, or data loss. Therefore, the number of players actively included in the analysis was limited to 20. All athletes had been actively engaged in football for at least three years, with a mean age of 22.25 ± 2.47 years and an average of 11.10 ± 5.07 years of sporting experience. The sample size was fixed, as it covered all available players from the team, and could not be increased during the study period; thus, *a priori* power analysis was not performed. However, repeated measurements taken from each participant over 14 weeks substantially increased the statistical power despite the relatively small sample size. Similarly, in longitudinal studies conducted at the single-team level in the literature (Curzi et al., 2024; Franks et al., 2025), the sample size was limited to the team roster, and no power analysis was performed.

### 2.2 Data collection instruments

For the purposes of this study, the Personal Information Form, Competitive Sport Anxiety Inventory-2 (CSAI-2), Visual Analogue Scale (VAS), Sport Emotion Questionnaire (SEQ), and Sport Psychological Recovery Scale (SPRS) were used to collect data on the study variables. Detailed information about the scales is provided below.

#### 2.2.1 Personal information form

The personal information form developed by the researcher consists of questions about the athletes’ demographic information, such as age, educational level, sports experience, and nationality, as well as the number of training sessions per week and the duration of each training session.

#### 2.2.2 CSAI-2

CSAI-2, developed by Martens et al. (1990a) to measure cognitive anxiety, physical anxiety, and self-confidence, was adapted into Turkish by Koruç (1998). The inventory consists of three subscales—cognitive anxiety, somatic anxiety, and self-confidence—and 27 items. Koruç (1998), examined the factor structure of the inventory and found that it had a three-factor structure, with these three factors explaining 78% of the total variance. Cronbach’s Alpha internal consistency coefficients for the subscales ranged from 0.74 to 0.89 (Koruç, 1998).

#### 2.2.3 VAS

The VAS is used to assess athletes’ subjective perceptions of the physical and mental difficulty experienced during competition (Wood et al., 1990). It has been widely used in previous sport-related studies to assess perceived exertion, fatigue, and psychological difficulty (Brown and Bray, 2019; Kayğusuz, 2024). During the administration, participants were asked to mark a point on a line between two endpoints that best reflected their perception. The left end of the scale was anchored as “1 = Almost no difficulty,” while the right end was anchored as “10 = Extremely difficult.” Participants indicated their perceived difficulty after the competition using this scale, following the instruction, “mark the difficulty level of your most recent competition.” This method allows for the assessment of how physically and mentally challenging the athlete found the competition to be.

#### 2.2.4 SEQ

The inventory developed by Jones et al. (2005) to measure athletes’ emotions during competition is completed either before or after the competition and was adapted into Turkish by Urfa and Aşçı (2019). The inventory, consisting of 22 emotion statements, is structured into five sub-dimensions: Anxiety, Dejection, Anger, Excitement, and Happiness. Athletes rate the emotion statements on a scale from 0 (Not at all) to 4 (Very much). Reliability studies for the scale indicate satisfactory reliability both before and after the competition (Cronbach’s alpha ranged from 0.70 to 0.89) (Jones et al., 2005; Allen et al., 2011). Furthermore, the fit indices obtained from confirmatory factor analysis support the construct validity of the scale (χ^2^/df = 1.38, GFI = 0.88, CFI = 0.95, RMR = 0.049, RMSEA = 0.04).

#### 2.2.5 SPRS

SPRS, developed by Kayğusuz and Karagözoğlu (2023), is used to measure athletes’ psychological recovery status after training or competition. The SPRS consists of 20 items and 4 subscales (Mental Recovery, Vigor and Energy, Psychological Detachment, and Flourishing). The measurement tool is scored on a 10-point Likert-type scale. The variance ratio explained in the EFA results was 69.88%. Also, CFA results indicated good model fit; regarding reliability, the internal consistency coefficients of the sub-dimensions were found to be 0.81 for Mental Recovery, 0.94 for Vigor and Energy, 0.83 for Psychological Detachment, and 0.93 for Flourishing (Kayğusuz and Karagözoğlu, 2023).

### 2.3 Data collection

At the beginning of data collection, during the pre-season preparation period, all participants were informed about the purpose, scope, and methodology of the research. The scientific nature of the study, the fact that the data obtained would be used solely for research purposes, and that all participant information would be protected in accordance with the principle of confidentiality were clearly stated. Participants were provided with detailed explanations about the research process, data collection times, and measurement tools to be used, and participation was voluntary. During this process, written consent was obtained in accordance with ethical principles, and all procedures were carried out.

It is noted that administering the CSAI-2 very shortly before the competition (e.g., 15–30 min) may disrupt athletes’ performance preparations and mental focus; it may also negatively affect the validity of the responses provided (Agaoglu, 2016; Aliberti et al., 2024). In contrast, administering the inventory approximately 1 h before the competition is recommended on the grounds that it reflects the athletes’ pre-performance anxiety level more accurately and allows for measurement without interfering with competition preparation routines (Craft et al., 2003; Muhammad et al., 2020; Hussain et al., 2021). Therefore, the CSAI-2 was administered one hour before the competition in this study. Similarly, it was preferred to administer the SEQ within the first hour after the competition. The literature indicates that emotions can change rapidly after the competition and may be encoded differently in memory over time, depending on cognitive reappraisal (Robinson and Clore, 2002; Uphill et al., 2014). Therefore, evaluating emotions as soon as possible after they are experienced increases the validity of the data obtained. Furthermore, given that emotions may fade rapidly or be supplanted by more enduring states, measuring them within the first 60 min after the competition allows for a more accurate and meaningful capture of the athletes’ immediate emotional responses (Allen et al., 2009). For these reasons, in this study, the SEQ was administered online within the first hour after the completion of each competition. Participants rated their overall perceived difficulty after the competition using the VAS. The VAS was administered online in a horizontal format. Studies have indicated that full recovery requires approximately 24–48 h (Bishop et al., 2008; González-Badillo et al., 2016; Morán-Navarro et al., 2017). In this context, considering the time required for athletes’ full recovery, the SPRS was administered online within 24–48 h post-competition.

The online data collection method made it possible to assess athletes’ immediate post-competition emotional states more quickly and effectively. All data collected throughout these processes were systematically recorded and secured for analysis in line with the overall objectives of the research. After all administrations were completed, the completed scales were reviewed for accuracy and completeness. Forms that were incomplete or incorrectly filled out were not evaluated; only scales of acceptable quality, defined as those with all items completed, consistent responses, and regular participation across the 14-week data collection period, were included in the analysis (Tables 1–6).

**Table 1:** Predictors of psychological recovery: cognitive anxiety and positive emotion.

Variable	*B*	SE	df	*t*	*p*
Intercept	6.33	0.46	274.23	13.76	0.001
Cognitive anxiety	−0.17	0.08	274.12	−2.16	0.032
Perceived difficulty	−0.12	0.05	268.43	−2.62	0.009
Positive emotion	0.43	0.05	269.14	8.53	0.001
Week	−0.04	0.01	257.17	−4.00	0.001

**Table 2:** Predictors of psychological recovery: somatic anxiety and positive emotion.

Variable	*B*	SE	df	*t*	*p*
Intercept	6.08	0.45	273.78	13.40	0.001
Somatic anxiety	−0.06	0.08	274.68	−0.79	0.430
Perceived difficulty	−0.14	0.05	268.62	−2.87	0.004
Positive emotion	0.46	0.05	268.57	9.06	0.001
Week	−0.05	0.01	256.95	−4.30	0.001

**Table 3:** Predictors of psychological recovery: self-confidence and positive emotion.

Variable	*B*	SE	df	*t*	*p*
Intercept	5.48	0.45	273.67	12.13	0.001
Self-confidence	0.19	0.07	272.77	2.69	0.008
Perceived difficulty	−0.13	0.05	266.65	−2.78	0.006
Positive emotion	0.42	0.05	267.53	8.42	0.001
Week	−0.05	0.01	256.48	−4.43	0.001

**Table 4:** Predictors of psychological recovery: cognitive anxiety and negative emotion.

Variable	*B*	SE	df	*t*	*p*
Intercept	8.74	0.30	273.72	29.14	0.001
Cognitive anxiety	−0.21	0.09	273.32	−2.43	0.016
Perceived difficulty	−0.23	0.05	269.95	−4.75	0.001
Negative emotion	−0.28	0.05	271.39	−5.41	0.004
Week	−0.05	0.01	257.42	−4.42	0.001

**Table 5:** Predictors of psychological recovery: somatic anxiety and negative emotion.

Variable	*B*	SE	df	*t*	*p*
Intercept	8.57	0.30	274.66	29.03	0.001
Somatic anxiety	−0.10	0.08	274.46	−1.21	0.228
Perceived difficulty	−0.24	0.05	270.80	−4.93	0.001
Negative emotion	−0.31	0.05	270.17	−6.08	0.001
Week	−0.05	0.01	256.89	−4.82	0.001

**Table 6:** Predictors of psychological recovery: self-confidence and negative emotions.

Variable	*B*	SE	df	*t*	*p*
Intercept	7.59	0.36	274.91	21.12	0.001
Self-confidence	0.26	0.07	273.43	3.63	0.001
Perceived difficulty	−0.24	0.05	268.43	−5.03	0.001
Negative emotion	−0.27	0.05	270.07	−5.39	0.002
Week	−0.05	0.01	256.41	−4.98	0.001

### 2.4 Statistical analysis

The data obtained in the study were analyzed using a linear mixed model (LMM) as they consisted of repeated measurements taken over a 14-week period. Recovery was modeled as the dependent variable. Pre-match anxiety, self-confidence, post-match negative and positive emotions, difficulties encountered, and the week variable were included in the analysis as fixed effects. To control for individual differences between participants, athletes were specified as random intercepts.

Model fit was evaluated using the Akaike Information Criterion (AIC), Bayesian Information Criterion (BIC), and log-likelihood values. Lower AIC and BIC values indicate better model fit when comparing alternative models; however, there are no absolute cut-off values for these indices (Burnham and Anderson, 2004). Furthermore, the model assumptions were examined; variance inflation factor (VIF) values below 5 were considered acceptable (Daoud, 2017), and visual inspection of residual plots indicated no major deviations from normality or homoscedasticity. All analyses were conducted in R (version 4.4.1; R Core Team, 2025) using the lme4 and lmerTest packages. The statistical significance level was set at *p* < 0.05.

## 3 Results

When examining the model results, cognitive anxiety (*B* = −0.17, *p* = 0.032) and perceived difficulty (*B* = −0.12, *p* = 0.009) showed a significant negative effect on psychological recovery. In contrast, positive emotion (*B* = 0.43, *p* < 0.001) emerged as a strong positive predictor. The week variable (*B* = −0.04, *p* < 0.001) also indicates a decreasing trend in recovery over time. The model’s explanatory power was high (R^2^m = 0.65; R^2^c = 0.69). The random effect variance associated with the athlete was found to be relatively low (ICC = 0.12), indicating that changes in psychological recovery are largely due to fixed effects, although individual differences also play a partial role. The model fit indices (AIC = 594.03, BIC = 619.47) were found to be acceptable.

When examining the model results, the encountered difficulty (*B* = −0.14, *p* = 0.004) had a significant negative effect on psychological recovery, while positive emotion (*B* = 0.46, *p* < 0.001) emerged as a strong positive predictor. The week variable (*B* = −0.05, *p* < 0.001) also indicated a decreasing trend in recovery over time. Physical anxiety, however, did not show a significant effect (*B* = −0.06, *p* = 0.430). The model’s explanatory power was high (R^2^m = 0.644; R^2^c = 0.685). The athlete-related random intercept variance was relatively low (ICC = 0.117), indicating that the variance in psychological recovery was largely due to fixed effects, but that individual differences also played a partial role. Model fit indices (AIC = 598.2, BIC = 623.6, REML = 584.2) were acceptable.

When examining the model results, self-confidence (*B* = 0.19, *p* = 0.008) and positive emotion (*B* = 0.42, *p* < 0.001) had a significant positive effect on psychological recovery. In contrast, the difficulty encountered (*B* = −0.13, *p* = 0.006) showed a significant negative effect. The week variable (*B* = −0.05, *p* < 0.001) also indicated a decreasing trend in recovery over time. The model’s explanatory power was high (R^2^m = 0.65; R^2^c = 0.70). The athlete-related random intercept variance was found to be relatively low (ICC = 0.14), indicating that the variance in psychological recovery was largely due to fixed effects, but that individual differences also played a partial role. The model fit indices (AIC = 588.69, BIC = 614.13, REML = 574.70) were acceptable.

When examining the model results, cognitive anxiety (*B* = −0.21, *p* = 0.016), perceived difficulty (*B* = −0.23, *p* < 0.001), and negative emotion (*B* = −0.28, *p* = 0.004) had a significant negative effect on psychological recovery. Furthermore, the week variable (*B* = −0.05, *p* < 0.001) also indicated a decreasing trend in recovery over time. The model’s explanatory power was high (R^2^m = 0.603; R^2^c = 0.646). Athlete-related random intercept variance was relatively low (ICC = 0.11), indicating that variance was largely due to fixed effects, but individual differences played a partial role. The model fit indices (AIC = 742.15, BIC = 768.40, REML = 729.88) were acceptable.

When examining the model results, the perceived difficulty (*B* = −0.24, *p* < 0.001) and negative emotion (*B* = −0.31, *p* < 0.001) showed a significant negative effect on psychological recovery. The week variable (*B* = −0.05, *p* < 0.001) also indicated a decreasing trend in recovery over time. Somatic anxiety, however, did not show a significant effect (*B* = −0.10, *p* = 0.228). The model’s explanatory power was high (R^2^m = 0.596; R^2^c = 0.639). The athlete-related random intercept variance was found to be low (ICC = 0.11), indicating that the variance in psychological recovery was largely due to fixed effects, but that individual differences also played a partial role. Model fit indices (AIC = 635.28, BIC = 660.72, REML = 621.3) were acceptable.

When examining the model results, it was observed that self-confidence had a significant positive effect on psychological recovery (*B* = 0.26, *p* < 0.001). In contrast, perceived difficulty (*B* = −0.24, *p* < 0.001) and negative emotion (*B* = −0.27, *p* < 0.002) were variables that negatively predicted psychological recovery. The week variable (*B* = −0.05, *p* < 0.001) also indicated a decreasing trend in recovery over time. The explanatory power of the model was high (R^2^m = 0.61; R^2^c = 0.66). The athlete-related random intercept variance was found to be relatively low (ICC = 0.13), indicating that the variance in psychological recovery was largely explained by fixed effects, but that individual differences were also contributed partially. The model fit indices (AIC = 624.12, BIC = 649.56, REML = 610.10) were acceptable.

## 4 Discussion

The high demands of sporting competitions demonstrate that not only training loads but also match outcomes can shape athletes’ emotional experiences, perceived levels of difficulty, and recovery processes (Kellmann, 2010; Hanin and Ekkekakis, 2014; Elbe et al., 2016). In this process, pre-competition levels of cognitive and somatic anxiety and self-confidence are important determinants of performance experiences and recovery capacity. However, competition losses or subpar performances can negatively affect athletes’ emotional experiences, performance self-evaluations, perceived difficulties, and recovery capacity. Conversely, successful performances or better-than-expected performances can facilitate the recovery process by strengthening positive emotions and self-confidence. In this context, recovery is not merely a physiological process; it is also a multidimensional process shaped by athletes’ emotional states and perceptual assessments. The aim of this study was to examine the role of anxiety dimensions, positive and negative emotions, and perceived difficulties in psychological recovery among athletes and to evaluate changes in recovery over time during the season. In the following discussion, the findings are discussed under headings corresponding to these variables.

### 4.1 The role of anxiety dimensions in psychological recovery

Our findings reveal that cognitive anxiety is one of the variables that negatively affects psychological recovery. Performance-related worries and negative thoughts weaken self-confidence and impede the recovery process by consuming the athlete’s mental resources. This finding is consistent with the predictions of the Multidimensional Anxiety Theory (Martens et al., 1990b), one of the fundamental theoretical models explaining the anxiety-performance relationship in the field of sports psychology. Similarly, Ford et al. (2017) found that the competitive sport-related anxiety can limit psychological recovery by disrupting attention processes and physiological arousal, particularly through anxiety-related cognitions. Furthermore, cognitive anxiety can negatively affect not only the immediate recovery process but also cognitive functions in subsequent competitions if recovery is inadequate. Indeed, the literature indicates that a lack of recovery leads to impairments in attention control, motivational processes, and self-regulation skills, thereby weakening performance readiness (Kellmann, 2002; Meeusen et al., 2013). Therefore, cognitive anxiety can be considered a critical risk factor that not only hinders short-term psychological recovery but also threatens the athletes’ performance readiness in the long term by affecting core cognitive and self-regulatory mechanisms.

Our study found that somatic anxiety does not have a significant effect on psychological recovery. is consistent with the literature, which suggests that somatic anxiety mostly reflects short-term and situation-specific physiological arousal and is not as decisive for long-term performance processes as cognitive anxiety (Ford et al., 2017). The Catastrophe Model of Hardy and Parfitt (1991) further elaborates on this interaction, showing that the effect of somatic anxiety depends on the level of cognitive anxiety. Indeed, Grossbard et al. (2009) reported in a study of young athletes that cognitive anxiety showed stronger correlations with performance, while somatic anxiety functioned as a more transient and situation-dependent dimension. Symptoms such as heart palpitations, muscle tension, or sweating experienced before training or competition can often be perceived by athletes as a natural part of preparing for performance (Thomas et al., 2007). Therefore, such physiological responses alone may not constitute a limiting factor in athletes’ psychological recovery capacity. Our findings also indicate that the effect of somatic anxiety on psychological recovery largely manifests through its interaction with cognitive evaluations. Therefore, it is important to focus not only on somatic symptoms but also on how athletes interpret these symptoms.

Our study found that self-confidence levels have a significant effect on psychological recovery. This finding shows that athletes’ confidence in themselves plays a decisive role not only in performance processes but also in the post-competition recovery processes. Recovery is defined as one of the central components of psychological resilience (Echezarraga et al., 2024). Resilience encompasses components such as self-confidence, optimism, and perseverance, and acts as a protective factor for individuals in coping with difficulties (Sisto et al., 2019). In this context, the central role of self-confidence in resilience suggests that it may support psychological recovery in athletes’ post-competition adjustment processes. Furthermore, as noted in the meta-analysis by Jekauc et al. (2025), self-confidence affects sports performance through mechanisms such as regulating performance under pressure, establishing psychological superiority over competitors, and using coping strategies more effectively. The association of these coping strategies with cognitive restructuring processes suggests that self-confidence may play a critical role not only during performance but also in accelerating psychological recovery after competition. These findings reveal that self-confidence is a multidimensional resource that influences both performance and psychological recovery due to its dynamic and comprehensive nature.

### 4.2 Emotional determinants of psychological recovery

The study findings indicate that positive emotions are one of the strongest predictors of psychological recovery. Studies in the context of sports psychology suggest that emotions generally change depending on training load (Millet et al., 2005; Meeusen et al., 2013; Martinent et al., 2014). The literature contains findings showing that emotional fluctuations are not only related to training periodization but are also significantly influenced by competition outcomes (Kerr et al., 2005; Sulu, 2024). Emotions exert both direct and indirect effects, not only as determinants of performance-related experiences but also in shaping recovery processes (Hanin and Ekkekakis, 2014). In this context, positive emotions are considered not merely the absence of negative emotions, but also a unique process that facilitates the construction, renewal, and recovery of psychological resources (Fredrickson and Levenson, 1998; Lundqvist and Kenttä, 2010).

On the other hand, the findings of our study indicate that negative emotions are a significant factor that weakens psychological recovery. Stress and negative emotions constrain athletes’ mental renewal capacity and slow the recovery process. Indeed, the literature contains many findings showing that athletes under high stress and negative emotions are associated with prolonged cardiovascular activation and slower physiological recovery processes (Pieper and Brosschot, 2005; Chida and Hamer, 2008). Furthermore, the close connection between persistent physiological activation and persistent cognitive processes (Brosschot et al., 2006) suggests that this process may also hinder psychological recovery. In this context, it has been reported that negative emotions are associated with a higher need for recovery and a lower level of psychological restructuring (Sonnentag and Zijlstra, 2006; Van Hooff et al., 2007). Similarly, it is emphasized that this process can negatively affect not only physiological recovery processes but also motivation for psychological renewal (Sonnentag et al., 2017). At the physiological level, it has been observed that competition results also reflect on emotional and recovery processes. In particular, winning has been found to be associated with an increase in testosterone, while losing has been associated with a decrease in testosterone (Geniole et al., 2017). These findings further reinforce the conclusion presented in our current study that positive emotions are the strongest predictors of recovery, while negative emotions weaken recovery and suggest that not only competition results but also training-related processes play a critical role in psychological recovery. Although training load was not directly measured in the present study, previous research suggests that the way athletes experience and regulate the psychological demands of training can significantly influence their emotional states and recovery quality (Kellmann, 2002; Kellmann et al., 2018). Structured training environments that promote balance between effort and recovery have been shown to enhance well-being and maintain adaptive emotional functioning across the season.

### 4.3 Perceived difficulty and time effect

The study found that an increase in perceived difficulty was negatively associated with recovery. Similar findings exist in the literature. It has been reported that an increase in perceived difficulty can hinder recovery processes and increase the risk of inadequate recovery and dysfunctional overloading (Kellmann, 2002; Meeusen et al., 2013). In this context, Saw et al. (2016) systematically reviewed studies demonstrating that the RESTQ-Sport (Recovery–Stress Questionnaire for Athletes) is sensitive to changes in training load; this sensitivity is particularly evident in the perceived difficulty dimension of internal load (sRPE); subjective recovery may weaken when load and accompanying perceived difficulty increase, but may improve when load is reduced. Furthermore, a review published by Kellmann et al. (2018) emphasizes that high perceived exertion can increase negative emotions in athletes and impair recovery. In a study conducted with elite swimmers during the four-month preparation period before the French swimming championship, an increase in anxiety, anger, and depressive mood over time was observed, while happiness and excitement decreased. These changes were reported to be related to the increased perceived difficulty and weakened recovery accompanying the approach of the competition period (Vacher et al., 2017). All these findings indicate that increased workload and psychological pressure throughout the season may have negative effects on recovery.

Findings related to the week variable reveal a declining trend in psychological recovery as the season progresses. Fluctuations in psychological recovery scores over the 14-week observation period may be attributed to athletes’ varying levels of anxiety, mood, and perceived difficulty. This trend is consistent with findings in the literature. For example, a study with female soccer players found that although training loads decreased in the later weeks of the season, recovery levels and neuromuscular performance fluctuated; it was observed that the increased load accumulation, especially in the later stages of the season, could weaken recovery capacity (Ishida et al., 2021). Similarly, Faude et al. (2011), found that professional soccer players experienced a decrease in recovery levels and an increase in stress scores throughout the season, with recovery processes becoming even more difficult toward the end of the season. These findings indicate that the cumulative effect of season-specific loading and psychological pressure can lead to fluctuations and a gradual decline in recovery capacity (Kenttä and Hassmén, 1998; Kellmann, 2002). The psychological recovery trend in our study is consistent with physiological indicators in the literature. Michailidis (2014), reported seasonal increases in cortisol and decreases in the testosterone/cortisol ratio in elite soccer players throughout the season; these fluctuations indicate physiological stress responses that can be linked to accumulated load and competitive demands. Curzi et al. (2024), observed decreases in testosterone levels and increases in cortisol levels in professional footballers during the second half of the season, suggesting that these hormonal changes reflect chronic loading and compromise athletes’ physiological recovery capacities toward the latter stages of the season. This finding highlights the importance of well-structured annual training plans that balance load and recovery across the competitive period. Previous research has emphasized that appropriate training load distribution can prevent chronic hormonal disturbances and lower the risk of overtraining (Issurin, 2010; Kiely, 2018). These findings indicate that not only psychological but also physiological recovery is compromised in the later stages of the season. Therefore, planning strategies to support recovery, especially in the later stages of the season is critical.

## 5 Limitations and future research

This study has some limitations. First, the findings are based solely on self-report scales, which may increase the risk of perceptual bias and common method variance. Psychological recovery is a subjective experience; the exclusion of behavioral indicators such as physiological stress responses, sleep, internal/external load, and coach/sports psychologist ratings limits the scope of interpretation. In future studies, multi-method designs combining these indicators with self-reports may reduce these risks. Second, limiting the sample to a specific group of athletes may restrict the generalizability of findings to other disciplines, competitive levels, and cultural contexts; future studies with larger samples, comparative designs, and measurement invariance tests could better capture contextual diversity. Finally, observational designs limit causal inferences; while findings suggest that cognitive anxiety, negative emotions, and self-confidence may be related to recovery, intervention-based research is required to establish causal directionality. Testing psychological skill programs aimed at reducing cognitive anxiety, regulating emotions, and strengthening self-confidence using experimental protocols may allow for the evaluation of causal effects on recovery and their transfer to performance.

## 6 Conclusion

This study indicates that psychological recovery is determined by a two-way pattern: cognitive anxiety, negative emotions, and perceived difficulty weaken recovery, while positive emotions and self-confidence strengthen it. The findings, consistent with the literature, suggest that interventions should focus not only on managing anxiety and negative emotions but also on systematically supporting self-confidence and positive emotions. In this regard, (i) mental training and cognitive reframing protocols, (ii) emotion regulation techniques, and (iii) the integration of post-competition psychological assessment and self-compassionate evaluation routines into training programs can play a critical role in maintaining performance and sustaining recovery capacity. These practices can meaningfully contribute to sustaining athletes’ optimal performance and recovery capacity, particularly in the later stages of the season. Thus, athletes can increase their likelihood of sustaining optimal performance levels by facilitating quicker recovery following perceived challenges.

## Data Availability

The datasets generated for this study are subject to restrictions due to participant confidentiality and ethical approval conditions. As the data were collected from a single team, individual-level information cannot be sufficiently anonymized and therefore cannot be shared publicly. Only aggregated results are presented in the manuscript. Requests to access the datasets should be directed to BS, busesulu@esenyurt.edu.tr.

## References

[ref1] Agaoglu Y. S. (2016). Responses to the competitive state anxiety inventory-2 by the athletes participated in the (IJF) judo grand prix competition, Samsun 2015 in Turkey. Int. J. Environ. Science Educ. 11, 3259–3275.

[ref2] Aliberti S. Raiola G. D’Elia F. Cherubini D. (2024). The influence of pre-competitive anxiety and self-confidence on Dancesport performance. Sports 12:308. doi: 10.3390/sports12110308, 39590910 PMC11598632

[ref3] Allen M. S. Jones M. V. Sheffield D. (2009). Causal attribution and emotion in the days following competition. J. Sports Sci. 27, 461–468. doi: 10.1080/02640410802538754, 19191165

[ref4] Allen M. S. Jones M. V. Sheffield D. (2011). Are the causes assigned to unsatisfactory performance related to the intensity of emotions experienced after competition. Sport Exercise Psychology Review 7, 3–10. doi: 10.53841/bpssepr.2011.7.1.3

[ref5] Barbosa-Granados S. Arenas-Granada J. Urrea H. García-Mas A. Reyes-Bossio M. Herrera-Velásquez D. . (2022). Precompetitive anxiety in young swimmers: analysis of the perceived competition difficulty (Ansiedad precompetitiva en nadadores juveniles: análisis desde la percepción del nivel de dificultad competitivo). Retos 45, 651–659. doi: 10.47197/retos.v45i0.90934

[ref6] Bishop P. A. Jones E. Woods A. K. (2008). Recovery from training: a brief review: brief review. J. Strength Cond. Res. 22, 1015–1024. doi: 10.1519/JSC.0b013e31816eb518, 18438210

[ref7] Brosschot J. F. Gerin W. Thayer J. F. (2006). The perseverative cognition hypothesis: a review of worry, prolonged stress-related physiological activation, and health. J. Psychosom. Res. 60, 113–124. doi: 10.1016/j.jpsychores.2005.06.074, 16439263

[ref8] Brown D. M. Bray S. R. (2019). Effects of mental fatigue on exercise intentions and behavior. Ann. Behav. Med. 53, 405–414. doi: 10.1093/abm/kay052, 29985969

[ref9] Burnham K. P. Anderson D. R. (2004). Multimodel inference: understanding AIC and BIC in model selection. Sociol. Methods Res. 33, 261–304. doi: 10.1177/0049124104268644

[ref10] Cerin E. (2003). Anxiety versus fundamental emotions as predictors of perceived functionality of pre-competitive emotional states, threat, and challenge in individual sports. J. Appl. Sport Psychol. 15, 223–238. doi: 10.1080/10413200305389

[ref11] Cerin E. Szabo A. Hunt N. Williams C. (2000). Temporal patterning of competitive emotions: a critical review. J. Sports Sci. 18, 605–626. doi: 10.1080/02640410050082314, 10972411

[ref12] Chida Y. Hamer M. (2008). Chronic psychosocial factors and acute physiological responses to laboratory-induced stress in healthy populations: a quantitative review of 30 years of investigations. Psychol. Bull. 134, 829–885. doi: 10.1037/a0013342, 18954159

[ref13] Cox R. (1998). Sport psychology: concepts and applications. Boston: McGraw-Hill.

[ref14] Craft L. L. Magyar T. M. Becker B. J. Feltz D. L. (2003). The relationship between the competitive state anxiety inventory-2 and sport performance: a meta-analysis. J. Sport Exerc. Psychol. 25, 44–65. doi: 10.1123/jsep.25.1.44

[ref15] Curzi D. Amatori S. Silvestri F. Marcelli L. Campanella M. Perroni F. . (2024). Professional football training and recovery: a longitudinal study on the effects of weekly conditioning session and workload variables. PLoS One 19:e0310036. doi: 10.1371/journal.pone.0310036, 39255308 PMC11386442

[ref16] Daoud J. I. (2017). “Multicollinearity and regression analysis”, in: 4th International Conference on Mathematical Applications in Engineering (ICMAE’17). (Kuala Lumpur, Malaysia: IOP Publishing).

[ref17] Davis A. J. Crittenden B. Cohen E. (2021). Effects of social support on performance outputs and perceived difficulty during physical exercise. Physiol. Behav. 239:113490. doi: 10.1016/j.physbeh.2021.113490, 34139269

[ref18] Echezarraga A. Las Hayas C. López de Arroyabe E. Jones S. H. (2024). Resilience and recovery in the context of psychological disorders. J. Humanist. Psychol. 64, 465–488. doi: 10.1177/0022167819851623

[ref19] Elbe A.-M. Rasmussen C. P. Nielsen G. Nordsborg N. B. (2016). High intensity and reduced volume training attenuates stress and recovery levels in elite swimmers. Eur. J. Sport Sci. 16, 344–349. doi: 10.1080/17461391.2015.1028466, 25867005

[ref20] Eysenck M. W. Derakshan N. Santos R. Calvo M. G. (2007). Anxiety and cognitive performance: attentional control theory. Emotion 7, 336–353. doi: 10.1037/1528-3542.7.2.336, 17516812

[ref21] Faude O. Kellmann M. Ammann T. Schnittker R. Meyer T. (2011). Seasonal changes in stress indicators in high level football. Int. J. Sports Med. 32, 259–265. doi: 10.1055/s-0030-1269894, 21271495

[ref22] FIFA (2019). “Women’s Football Member Associations Survey Report”. (Zurich, Switzerland).

[ref23] Fletcher D. Scott M. (2010). Psychological stress in sports coaches: a review of concepts, research, and practice. J. Sports Sci. 28, 127–137. doi: 10.1080/02640410903406208, 20035490

[ref24] Ford J. L. Ildefonso K. Jones M. L. Arvinen-Barrow M. (2017). Sport-related anxiety: current insights. Open Access J. Sports Med. 8, 205–212. doi: 10.2147/oajsm.S125845 29138604 PMC5667788

[ref25] Fox E. (2008). Emotion science cognitive and neuroscientific approaches to understanding human emotions. New York: Palgrave Macmillan.

[ref26] Franks C. Barkesdale L. Armstrong K. Jo J. Myers T. Andre T. . (2025). Does load matter? The effects of training load on injury and illness in division I female soccer players. Research Strength Performance 5, 1–6.

[ref27] Fredrickson B. Levenson R. W. (1998). Positive emotions speed recovery from the cardiovascular sequelae of negative emotions. Cogn. Emot. 12, 191–220. doi: 10.1080/026999398379718, 21852890 PMC3156608

[ref28] Geniole S. N. Bird B. M. Ruddick E. L. Carré J. M. (2017). Effects of competition outcome on testosterone concentrations in humans: an updated meta-analysis. Horm. Behav. 92, 37–50. doi: 10.1016/j.yhbeh.2016.10.002, 27720891

[ref29] González-Badillo J. J. Rodríguez-Rosell D. Sánchez-Medina L. Ribas J. López-López C. Mora-Custodio R. . (2016). Short-term recovery following resistance exercise leading or not to failure. Int. J. Sports Med. 37, 295–304. doi: 10.1055/s-0035-1564254 26667923

[ref30] Gross J. J. (1998). The emerging field of emotion regulation: an integrative review. Rev. Gen. Psychol. 2, 271–299. doi: 10.1037/1089-2680.2.3.271

[ref31] Grossbard J. R. Smith R. E. Smoll F. L. Cumming S. P. (2009). Competitive anxiety in young athletes: differentiating somatic anxiety, worry, and concentration disruption. Anxiety Stress Coping 22, 153–166. doi: 10.1080/10615800802020643, 18937102

[ref33] Hanin Y. (2000). “Individual zones of optimal functioning (IZOF) model,” in Emotions in Sport. (Champaign, IL: Human Kinetics).

[ref34] Hanin J. Ekkekakis P. (2014). “Emotions in sport and exercise settings,” in Routledge companion to sport and exercise psychology. (London/New York: Routledge), 83–104.

[ref35] Hardy L. Parfitt G. (1991). A catastrophe model of anxiety and performance. Br. J. Psychol. 82, 163–178. doi: 10.1111/j.2044-8295.1991.tb02391.x, 1873650

[ref36] Harrison K. Potts E. King A. C. Braun-Trocchio R. (2021). The effectiveness of virtual reality on anxiety and performance in female soccer players. Sports 9:167. doi: 10.3390/sports9120167, 34941805 PMC8703795

[ref37] Höner O. Feichtinger P. (2016). Psychological talent predictors in early adolescence and their empirical relationship with current and future performance in soccer. Psychol. Sport Exerc. 25, 17–26. doi: 10.1016/j.psychsport.2016.03.004

[ref38] Hussain F. Shah M. Ali A. (2021). Analysis of sports anxiety levels among experienced and inexperienced collegiate athletes. Res. J. Soc. Sci. Econ. Rev. 2, 460–465. doi: 10.36902/rjsser-vol2-iss1-2021(460-465)

[ref39] Ishida A. Bazyler C. D. Sayers A. L. Stone M. H. Gentles J. A. (2021). Seasonal changes and relationships in training loads, neuromuscular performance, and recovery and stress state in competitive female soccer players. Front. Sports Active Living 3:757253. doi: 10.3389/fspor.2021.757253, 34708201 PMC8542871

[ref40] Issurin V. B. (2010). New horizons for the methodology and physiology of training periodization. Sports Med. 40, 189–206. doi: 10.2165/11319770-000000000-00000, 20199119

[ref41] Janelle C. M. Fawver B. J. Beatty G. F. (2020). “Emotion and sport performance,” in Handbook of sport psychology, eds. G. Tenenbaum & R.C. Eklund. 4th ed. (Hoboken, NJ: Wiley).

[ref42] Jekauc D. Fiedler J. Wunsch K. Muelberger L. Burkart D. Kilgus A. . (2025). The effect of self-confidence on performance in sports: a meta-analysis and narrative review. Int. Rev. Sport Exerc. Psychol. 18, 345–371. doi: 10.1080/1750984X.2023.2222376

[ref43] Jones M. V. Lane A. M. Bray S. R. Uphill M. Catlin J. (2005). Development and validation of the sport emotion questionnaire. J. Sport Exerc. Psychol. 27, 407–431. doi: 10.1123/jsep.27.4.407

[ref44] Kayğusuz Ş. (2024). A longitudinal study on the relationship of mental fatigue with rest and psychological recovery in team athletes. Doctoral dissertation: Marmara University.

[ref45] Kayğusuz Ş. Karagözoğlu C. (2023). Sporda psikolojik toparlanma ölçeği (SPTÖ) geliştirme çalışması. Spor Bilimleri Araştırmaları Dergisi 8, 157–174. doi: 10.25307/jssr.1192861

[ref46] Kellmann M. (2002). Enhancing recovery: Preventing underperformance in athletes. Champaign, IL: Human Kinetics.

[ref47] Kellmann M. (2010). Preventing overtraining in athletes in high-intensity sports and stress/recovery monitoring. Scand. J. Med. Sci. Sports 20, 95–102. doi: 10.1111/j.1600-0838.2010.01192.x, 20840567

[ref48] Kellmann M. Bertollo M. Bosquet L. Brink M. Coutts A. J. Duffield R. . (2018). Recovery and performance in sport: consensus statement. Int. J. Sports Physiol. Perform. 13, 240–245. doi: 10.1123/ijspp.2017-0759, 29345524

[ref49] Kellmann M. Kallus K. W. (1999). Recovery-stress questionnaire for athletes: User manual. Champaign, IL: Human Kinetics.

[ref50] Kenttä G. Hassmén P. (1998). Overtraining and recovery: a conceptual model. Sports Med. 26, 1–16. doi: 10.2165/00007256-199826010-00001, 9739537

[ref51] Kerr J. H. Wilson G. V. Nakamura I. Sudo Y. (2005). Emotional dynamics of soccer fans at winning and losing games. Personal. Individ. Differ. 38, 1855–1866. doi: 10.1016/j.paid.2004.10.002

[ref52] Keyes C. L. (2002). The mental health continuum: from languishing to flourishing in life. J. Health Soc. Behav. 43, 207–222. doi: 10.2307/3090197 12096700

[ref53] Kiely J. (2018). Periodization theory: confronting an inconvenient truth. Sports Med. 48, 753–764. doi: 10.1007/s40279-017-0823-y, 29189930 PMC5856877

[ref54] Koruç Z. (1998). CSAI-2’nin Türkiye uyarlaması, ön çalışma I. Spor Bilimleri V. Kongresi, Hacettepe Üniversitesi, Ankara, 93.

[ref55] Lane A. M. Terry P. C. (2000). The nature of mood: development of a conceptual model with a focus on depression. J. Appl. Sport Psychol. 12, 16–33. doi: 10.1080/10413200008404211

[ref56] Lazarus R. S. (2000). How emotions influence performance in competitive sports. Sport Psychol. 14, 229–252. doi: 10.1123/tsp.14.3.229

[ref57] Lovell R. Okholm Kryger K. (2025). Back to the future—past learnings for prospective performance, medicine and Health Research recommendations in WOMEN’s football: future directions in women’s football research: the WOMEN framework. Sports Med., 1–9. doi: 10.1007/s40279-025-02250-1 40853528 PMC13314819

[ref58] Lundqvist C. (2011). Well-being in competitive sports—the feel-good factor? A review of conceptual considerations of well-being. Int. Rev. Sport Exerc. Psychol. 4, 109–127. doi: 10.1080/1750984X.2011.584067

[ref59] Lundqvist C. Kenttä G. (2010). Positive emotions are not simply the absence of the negative ones: development and validation of the emotional recovery questionnaire (EmRecQ). Sport Psychol. 24, 468–488. doi: 10.1123/tsp.24.4.468

[ref60] Mabweazara S. Z. Leach L. Andrews B. S. (2017). Predicting swimming performance using state anxiety. S. Afr. J. Psychol. 47, 110–120. doi: 10.1177/0081246316645060

[ref61] Madsen E. E. Hansen T. Thomsen S. D. Panduro J. Ermidis G. Krustrup P. . (2022). Can psychological characteristics, football experience, and player status predict state anxiety before important matches in Danish elite-level female football players? Scand. J. Med. Sci. Sports 32, 150–160. doi: 10.1111/sms.13881, 33202060

[ref62] Martens R. Burton D. Vealey R. S. Bump L. A. Smith D. E. (1990a). Development and validation of the competitive state anxiety inventory-2. Competitive Anxiety Sport 3, 117–190.

[ref63] Martens R. Vealey R. S. Burton D. (1990b). Competitive anxiety in sport. Champaign, IL: Human Kinetics.

[ref64] Martinent G. Campo M. Ferrand C. (2012). A descriptive study of emotional process during competition: nature, frequency, direction, duration and co-occurrence of discrete emotions. Psychol. Sport Exerc. 13, 142–151. doi: 10.1016/j.psychsport.2011.10.006

[ref65] Martinent G. Decret J.-C. Filaire E. Isoard-Gautheur S. Ferrand C. (2014). Evaluations of the psychometric properties of the recovery-stress questionnaire for athletes among a sample of young French table tennis players. Psychol. Rep. 114, 326–340. doi: 10.2466/03.14.PR0.114k18w2, 24897893

[ref66] Martinent G. Ferrand C. (2009). A naturalistic study of the directional interpretation process of discrete emotions during high-stakes table tennis matches. J. Sport Exerc. Psychol. 31, 318–336. doi: 10.1123/jsep.31.3.318, 19798996

[ref68] Meeusen R. Duclos M. Foster C. Fry A. Gleeson M. Nieman D. . (2013). Prevention, diagnosis and treatment of the overtraining syndrome: joint consensus statement of the European College of Sport Science (ECSS) and the American College of Sports Medicine (ACSM). Eur. J. Sport Sci. 13, 1–24. doi: 10.1080/17461391.2012.730061 23247672

[ref69] Michailidis Y. (2014). Stress hormonal analysis in elite soccer players during a season. J. Sport Health Sci. 3, 279–283. doi: 10.1016/j.jshs.2014.03.016

[ref70] Millet G. Groslambert A. Barbier B. Rouillon J. Candau R. (2005). Modelling the relationships between training, anxiety, and fatigue in elite athletes. Int. J. Sports Med. 26, 492–498. doi: 10.1055/s-2004-821137, 16037894

[ref71] Morán-Navarro R. Pérez C. E. Mora-Rodríguez R. de la Cruz-Sánchez E. González-Badillo J. J. Sánchez-Medina L. . (2017). Time course of recovery following resistance training leading or not to failure. Eur. J. Appl. Physiol. 117, 2387–2399. doi: 10.1007/s00421-017-3725-7, 28965198

[ref67] Muhammad N. Khan M. Khan W. (2020). Effect of different types of Anxiety on Athletes Performance; Planning and Managing Strategy to cope with Athletes’ Anxiety. City University Research Journal, 10, 471–486.

[ref72] Pelka M. Kölling S. Ferrauti A. Meyer T. Pfeiffer M. Kellmann M. (2017). Acute effects of psychological relaxation techniques between two physical tasks. J. Sports Sci. 35, 216–223. doi: 10.1080/02640414.2016.1161208, 26999625

[ref73] Pieper S. Brosschot J. F. (2005). Prolonged stress-related cardiovascular activation: is there any? Ann. Behav. Med. 30, 91–103. doi: 10.1207/s15324796abm3002_1, 16173905

[ref74] Randell R. K. Clifford T. Drust B. Moss S. L. Unnithan V. B. De Ste Croix M. B. . (2021). Physiological characteristics of female soccer players and health and performance considerations: a narrative review. Sports Med. 51, 1377–1399. doi: 10.1007/s40279-021-01458-1, 33844195 PMC8222040

[ref75] Reilly T. Williams A. M. Nevill A. Franks A. (2000). A multidisciplinary approach to talent identification in soccer. J. Sports Sci. 18, 695–702. doi: 10.1080/02640410050120078, 11043895

[ref76] Robinson M. D. Clore G. L. (2002). Belief and feeling: evidence for an accessibility model of emotional self-report. Psychol. Bull. 128, 934–960. doi: 10.1037/0033-2909.128.6.934, 12405138

[ref77] Rocha V. V. S. Osorio F. D. L. (2018). Associations between competitive anxiety, athlete characteristics and sport context: evidence from a systematic review and meta-analysis. Archives Clinical Psychiatry 45, 67–74. doi: 10.1590/0101-60830000000160

[ref78] Ryan R. M. Frederick C. (1997). On energy, personality, and health: subjective vitality as a dynamic reflection of well-being. J. Pers. 65, 529–565. doi: 10.1111/j.1467-6494.1997.tb00326.x, 9327588

[ref79] Salom Martorell M. Ponseti F. J. Núñez Prats A. Contestí Bosch B. Salom Ferragut G. García-Mas A. (2021). Competitive anxiety and performance in competing sailors. Retos 39, 187–191.

[ref80] Sánchez-Ruiz R. Gil-Caselles L. García-Naveira A. Arbinaga F. Ruiz-Barquín R. Olmedilla-Zafra A. (2025). Competitive anxiety, sports injury, and playing category in youth soccer players. Children 12:1094. doi: 10.3390/children12081094, 40868545 PMC12384764

[ref81] Saw A. E. Main L. C. Gastin P. B. (2016). Monitoring the athlete training response: subjective self-reported measures trump commonly used objective measures: a systematic review. Br. J. Sports Med. 50, 281–291. doi: 10.1136/bjsports-2015-094758, 26423706 PMC4789708

[ref82] Seligman M. E. (2011). Flourish: A visionary new understanding of happiness and well-being. New York: Simon and Schuster.

[ref83] Sisto A. Vicinanza F. Campanozzi L. L. Ricci G. Tartaglini D. Tambone V. (2019). Towards a transversal definition of psychological resilience: a literature review. Medicina 55:745. doi: 10.3390/medicina55110745, 31744109 PMC6915594

[ref84] Sonnentag S. Fritz C. (2007). The recovery experience questionnaire: development and validation of a measure for assessing recuperation and unwinding from work. J. Occup. Health Psychol. 12, 204–221. doi: 10.1037/1076-8998.12.3.204, 17638488

[ref85] Sonnentag S. Venz L. Casper A. (2017). Advances in recovery research: what have we learned? What should be done next? J. Occup. Health Psychol. 22, 365–380. doi: 10.1037/ocp0000079, 28358572

[ref86] Sonnentag S. Zijlstra F. R. (2006). Job characteristics and off-job activities as predictors of need for recovery, well-being, and fatigue. J. Appl. Psychol. 91, 330–350. doi: 10.1037/0021-9010.91.2.330, 16551187

[ref87] Sulu B. 2024 Predictors of the direction and intensity of competition anxiety in female volleyball players. PhD thesis/Doctoral dissertation, Ankara University

[ref88] Tamir M. Mitchell C. Gross J. J. (2008). Hedonic and instrumental motives in anger regulation. Psychol. Sci. 19, 324–328. doi: 10.1111/j.1467-9280.2008.02088.x, 18399883

[ref89] Tamminen K. A. Gaudreau P. (2014). “Coping, social support, and emotion regulation in teams” in Group dynamics in exercise and sport psychology. (Eds.) Beauchamp, M. R., and Eys, M. A. (New York: Routledge), 222–239.

[ref90] Thomas O. Hanton S. Maynard I. (2007). Psychological preparation for elite performers: the role of interpretation of competitive anxiety symptoms. Sport Psychol. 21, 473–494. doi: 10.1123/tsp.21.4.473

[ref91] Tossici G. Zurloni V. Nitri A. (2024). Stress and sport performance: a PNEI multidisciplinary approach. Front. Psychol. 15:1358771. doi: 10.3389/fpsyg.2024.1358771, 38495423 PMC10940545

[ref92] Uphill M. Groom R. Jones M. (2014). The influence of in-game emotions on basketball performance. Eur. J. Sport Sci. 14, 76–83. doi: 10.1080/17461391.2012.729088, 24533498

[ref93] Urfa O. Aşçı F. H. (2019). Spor Duygu Ölçeği: Geçerlik Ve Güvenirlik Çalişmasi. Spormetre Beden Eğitimi ve Spor Bilimleri Dergisi 17, 42–55. doi: 10.33689/spormetre.518329

[ref94] Vacher P. Nicolas M. Martinent G. Mourot L. (2017). Changes of swimmers' emotional states during the preparation of National Championship: do recovery-stress states matter? Front. Psychol. 8:1043. doi: 10.3389/fpsyg.2017.01043, 28690573 PMC5481396

[ref95] Van Hooff M. L. Geurts S. A. Kompier M. A. Taris T. W. (2007). “How fatigued do you currently feel?” convergent and discriminant validity of a single-item fatigue measure. J. Occup. Health 49, 224–234. doi: 10.1539/joh.49.224, 17575403

[ref96] Wood C. Magnello M. Jewell T. (1990). Measuring vitality. J. R. Soc. Med. 83, 486–489. doi: 10.1177/014107689008300803, 2231574 PMC1292771

[ref97] Woodman T. Hardy L. (2003). The relative impact of cognitive anxiety and self-confidence upon sport performance: a meta-analysis. J. Sports Sci. 21, 443–457. doi: 10.1080/0264041031000101809, 12846532

